# Frizzled receptors: gatekeepers of Wnt signaling in development and disease

**DOI:** 10.3389/fcell.2025.1599355

**Published:** 2025-05-01

**Authors:** Dalia Martinez-Marin, Grace C. Stroman, Camryn J. Fulton, Kevin Pruitt

**Affiliations:** Department of Pharmacology, University of North Carolina at Chapel Hill, Chapel Hill, NC, United States

**Keywords:** Wnt, cancer, FZD, frizzled receptor, signaling, DVL

## Abstract

Frizzled (FZD) receptors are a subset of G-protein-coupled receptors (GPCRs), the largest class of human cell surface receptors and a major target of FDA-approved drugs. Activated by Wnt ligands, FZDs regulate key cellular processes such as proliferation, differentiation, and polarity, positioning them at the intersection of developmental biology and disease, including cancer. Despite their significance, FZD signaling remains incompletely understood, particularly in distinguishing receptor-specific roles across canonical and non-canonical Wnt pathways. Challenges include defining ligand-receptor specificity, elucidating signal transduction mechanisms, and understanding the influence of post translational modifications and the cellular context. Structural dynamics, receptor trafficking, and non-canonical signaling contributions also remain areas of active investigation. Recent advances in structural biology, transcriptomics, and functional genomics are beginning to address these gaps, while emerging therapeutic approaches—such as small-molecule modulators and antibodies—highlight the potential of FZDs as drug targets. This review synthesizes current insights into FZD receptor biology, examines ongoing controversies, and outlines promising directions for future research and therapeutic development.

## Introduction

Frizzled (FZD) receptors belong to the family of G-protein-coupled receptors (GPCRs), the largest group of cell surface receptors in humans (Schulte and Bryja, 2007; Dijksterhuis et al., 2015), where 35% of FDA-approved drugs target GPCRs (Sriram and Insel, 2018). FZDs are activated by the binding of Wnt ligands which regulate critical processes like cell proliferation, differentiation, and polarity. These receptors are central to developmental biology and disease pathology but are not without controversy. The mechanisms of FZD activation, receptor-specific roles in canonical (Wnt/β-catenin) versus non-canonical [Wnt/Ca^2+^, planar cell polarity (PCP)] signaling, and their interactions with co-receptors and signaling networks remain unclear (Huang and Klein, 2004; Zhan et al., 2017). These divergent models reflect the complexity and context-dependent nature of FZD function, underscoring the need for further study. Fundamental questions in FZD biology include how ligand-receptor specificity is determined, the mechanisms of signal transduction, and the regulatory roles of post-translational modifications and the cellular environment. Key challenges remain in understanding the structural basis of receptor-ligand interactions, the dynamics of receptor trafficking, and the distinct contributions of non-canonical Wnt signaling in health and disease. Addressing these gaps is crucial for developing targeted therapies for conditions like cancer, neurodegeneration, and developmental disorders. Recent advances in structural biology, transcriptomics, and functional genomics are beginning to shed light on these unanswered questions. Emerging therapeutic strategies, including small-molecule modulators and antibody-based approaches, offer promising avenues for targeting FZD receptors. This review evaluates current knowledge of FZD receptor biology, addressing controversies, unresolved issues, and research gaps while exploring recent advances and future directions in this rapidly evolving field.

## Structural characteristics of FZD receptors

**FIGURE 1:**
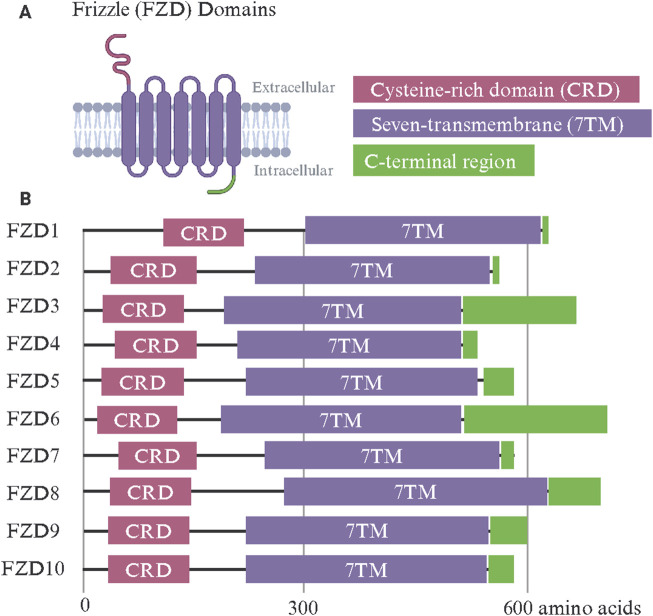
Frizzle (FZD) receptor family protein domains. **(A)** The FZD receptor family shares a common set of functional domains critical for Wnt binding and intracellular signal transmission. The extracellular cysteine-rich domain (CRD) confers Wnt ligand specificity. A seven-transmembrane (7TM) domain is shared among G-protein-coupled receptors and transmits Wnt binding through conformational changes. The intracellular C-terminal region includes a PDZ-binding motif and recruits intracellular signaling effectors such as Dishevelled. **(B)** The linear protein domains of human FZD1-10. Made with Biorender.com.

The FZD receptor family includes ten members (FZD1–FZD10), each exhibiting a conserved modular design tailored for binding Wnt ligands (Schulte, 2010) (Figure 1). FZDs belong to class F GPCRs and these receptors share a common architecture, including an extracellular cysteine-rich domain (CRD), a hydrophilic linker region, a seven-transmembrane (7TM) domain, and an intracellular C-terminal region. Subtle structural differences among family members confer specificity in ligand binding, signaling, and function (Janda et al., 2012; Agostino and Pohl, 2019). The extracellular CRD is a hallmark feature of all FZD receptors and is critical for binding Wnt ligands (Egger-Adam and Katanaev, 2008). This domain is stabilized by ten conserved cysteine residues that form disulfide bonds, creating a structure that interacts with the lipid-modified regions of Wnt ligands, such as palmitoleic acid (Chong et al., 2002a). Despite being highly conserved, variations in the CRD sequence between different FZD receptors could influence their selectivity for Wnt ligands (Li N. et al., 2019; Kowalski-Jahn et al., 2021). This specificity is critical for directing signaling and receptor function (Milhem and Ali, 2019; Grätz et al., 2023). The 7TM domain, typical of GPCRs, anchors the FZD receptors in the plasma membrane and facilitates the transduction of extracellular signals into intracellular responses (Zhang et al., 2017). Unlike classical GPCRs, FZD receptors interact less frequently with heterotrimeric G-proteins, relying instead on other intracellular signaling proteins, such as Dishevelled (DVL) (Seitz et al., 2014; Zhan et al., 2017). Variability in the 7TM domain among FZD receptors likely contributes to their ability to activate specific downstream pathways and may explain their differential involvement in the different branches of Wnt signaling (Carron et al., 2003; Tesmer, 2016). The linker domain, which connects the CRD and 7TM domains, has been indicated in signal specificity. For example, when the linker domain of FZD4 was replaced with that of FZD6, Wnt3a signaling was diminished (Ko et al., 2022). Despite its importance, the linker domain remains understudied due to its flexible nature and inability to be resolved in structures. The intracellular C-terminal region, which often includes a PDZ-binding motif, is key in recruiting signaling effectors like DVL (Tauriello et al., 2012; Liu et al., 2022). While the key post-translationally modified residues within conserved domains of DVL, such as the PDZ domain, have been shown to regulate its nuclear translocation (Sharma et al., 2019; Sharma et al., 2021), little is known about what controls DVL trafficking to the plasma membrane vs the nucleus (Boligala et al., 2022). This region is subject to post-translational modifications, such as phosphorylation, which regulate receptor activity, trafficking, and recycling (Pascual-Vargas and Salinas, 2021).

FZD1 and FZD2 have highly conserved CRDs with a characteristic arrangement of disulfide bonds, stabilizing the domain for efficient Wnt ligand binding (Pourreyron et al., 2012; Agostino et al., 2017). Both receptors interact primarily with canonical Wnt ligands such as Wnt3a and Wnt1 (Laeremans et al., 2011). Their 7TM domains exhibit typical GPCR features, with minor variations in extracellular loops that may affect ligand specificity (Zeng C. M. et al., 2018). Their intracellular C-terminal regions include PDZ-binding motifs essential for recruiting DVL and contain phosphorylation sites that regulate receptor activity and recycling. FZD2 exhibits broad ligand affinity, attributed to subtle differences in the ligand-binding pocket of its CRD (Agostino et al., 2017). FZD3 and FZD6 are primarily associated with non-canonical signaling pathways (Pourreyron et al., 2012; Corda and Sala, 2017). One study compared the FZD3 palmitoleic moiety groove in the CRD and found that FZD3’s was narrower compared to that of FZD8, though this could be due in part to the binding of a nanobody (Hillier et al., 2024). Their CRDs are adapted for binding non-canonical Wnt ligands such as Wnt5a and Wnt11 (Li C. et al., 2019). Compared to FZD1 and FZD2, their 7TM domains display unique loop structures, contributing to their preference for PCP signaling (Tophkhane et al., 2024; Zhang et al., 2024b). Notably, FZD6 lacks a PDZ-binding motif in its intracellular C-terminal domain, setting it apart from most other FZD receptors and influencing its distinct signaling behavior in tissue polarity and morphogenesis (Lyons et al., 2004). FZD4 features a structurally distinct CRD that facilitates its interaction with the atypical ligand Norrin, as well as Wnt7a and Wnt7b (Wang et al., 2018). This receptor is critical in vascular development, particularly in the retina (Xu et al., 2004). Its 7TM domain has unique residues that support its specific signaling activity, and its C-terminal domain includes a PDZ-binding motif that interacts with effector proteins (Zhang et al., 2011). Mutations in the CTD of FZD4, that encompasses the K-T/S-XXX-W, a PDZ binding motif and S/T-X-V PDZ recognition motif, are associated with familial exudative vitreoretinopathy (FEVR), underscoring the functional importance of its unique structural features (Kondo et al., 2003; Fei et al., 2015; Seemab et al., 2019). Deletion mutations of FZD4 residues such as L420, L430, L433, and W335 were also shown to attenuate interaction with the DEP domain of DVL (Qian et al., 2024a). FZD5 shares similarities with FZD4 in its ability to bind Wnt5a and Wnt7a but has a broader expression profile during embryogenesis (Ishikawa et al., 2001; Burns et al., 2008; Carmon and Loose, 2008). Its CRD and 7TM domains are well-adapted to mediate Wnt signaling pathways, with a structurally conserved C-terminal domain facilitating downstream interactions (Cavodeassi et al., 2005; Liu and Nathans, 2008).

FZD7 is the most well-studied paralog in cancer and its overexpression has been documented across numerous tumor types. Recent cryo-electron microscopy (cryo-EM) studies by Schulte and colleagues have reported the structure of inactive FZD7 without any stabilizing mutations (Bous et al., 2024). They further identify lipids interacting with the FZD7 receptor core and a conserved cholesterol-binding site which is key in mediating DVL-FZD7 association and downstream signaling. FZD8 is closely related to FZD5 in structure, with a CRD optimized for Wnt3a binding (Voloshanenko et al., 2017; Hua et al., 2018). It's frequently implicated in cancer due to its involvement in cell proliferation and differentiation (Sun et al., 2020; Dong et al., 2021). FZD9 is characterized by a CRD with unique sequence features that may influence its specificity for Wnt2 and Wnt5a ligands (Karasawa et al., 2002; Ramírez et al., 2016). It is predominantly expressed in the central nervous system, with a 7TM domain and C-terminal region resembling canonical FZDs but specialized for neurodevelopmental processes (Wang et al., 1997; Wang Y.-K. et al., 1999; Ramírez et al., 2016). Mutations in FZD9 have been linked to Williams-Beuren syndrome, suggesting a role for its specific structural elements in neural connectivity (Wang Y. K. et al., 1999). FZD10 stands out for its overexpression in cancers such as synovial sarcoma and hepatocellular carcinoma (Nagayama et al., 2005; Wang et al., 2023). Its CRD is highly efficient at binding Wnt1 and Wnt3a, driving robust canonical Wnt/β-catenin signaling (Galli et al., 2014; Alrefaei et al., 2020). Its 7TM and C-terminal domains are well-adapted for signal amplification, with phosphorylation sites and a PDZ-binding motif that enhance interactions with DVL (Fukukawa et al., 2009; Hot et al., 2017). Unlike other FZD receptors, FZD10 is minimally expressed in normal tissues, highlighting its potential as a therapeutic target.

The CRD’s role in Wnt specificity and binding remains a focus of debate. While it is widely accepted as the primary binding site for Wnt ligands, alternative models suggest that co-receptors, such as LRP5/6, or the lipid bilayer environment, may modulate this interaction. Similarly, the exact contributions of the 7TM domain to signal transduction are contentious. Unlike classical GPCRs, FZD receptors exhibit limited direct G-protein coupling, leading to debates over whether they should be classified strictly as GPCRs or as part of a broader receptor family with hybrid functionalities. Key questions in FZD receptor biology include how structural elements coordinate to ensure pathway specificity and how post-translational modifications regulate receptor activity. The role of the intracellular C-terminal domain in engaging diverse Wnt signaling pathways, also raises critical issues. Furthermore, the mechanistic basis for crosstalk between FZD receptors and other signaling networks remains poorly understood.

Recent advancements in the study of FZD receptors have been propelled by innovations in structural biology, imaging technologies, functional genomics, and computational modeling, significantly enhancing our understanding of their role in Wnt signaling, development, and disease. High-resolution structural studies, particularly through cryo-EM, have provided unprecedented insights into the architecture of FZD receptors and their interactions with Wnt ligands, co-receptors, and intracellular effectors. Cryo-EM has revealed the precise binding of lipid-modified Wnt ligands, such as the palmitoleic acid moiety on Wnt3a, to the CRD of FZD receptors, uncovering the structural basis of ligand specificity (Tsutsumi et al., 2020; Xu et al., 2021a; Zhong et al., 2021; Qian et al., 2024b). These studies have also captured conformational changes in the 7TM domain of FZD receptors, elucidating how extracellular ligand binding induces intracellular signaling cascades. Comparison of apo vs. G protein-bound cryo-EM structures revealed that TM7 of FZD1 shifts inward upon activation, which was not shown in FZD3 and FZD6 structures. Additionally, FZD3 and FZD6 displayed more inward shifts of TM5 compared to FZD1 (Zhang et al., 2024a). This dynamic structural information could potentially be leveraged to modulate specific intracellular pathways. Complementing cryo-EM, computational modeling, and molecular dynamics simulations have provided dynamic views of receptor activation, including the influence of lipid bilayer environments on receptor function and the allosteric mechanisms by which ligand binding propagates structural changes to intracellular signaling domains. In FZD7, distinct conformations between interacting residues W354-Y478 and R470-W547 show characteristic rotamer flips when comparing the active (G protein-bound) and inactive (apo) cryo-EM structures. Molecular dynamic simulations indicated further evidence that these movements are a permanent switch that occurs upon G protein interaction, rather than a transient state captured by cryo-EM. (https://www.nature.com/articles/s41467-024-51664-4). Molecular dynamics experiments have also predicted unique interactions such as Wnt2 freely turning around FZD7 CRD, and decreased β-strand formation upon binding to the CRD (Kalhor et al., 2018).

Despite advancements in structural biology, high-resolution models of FZD receptors in complex with their ligands and co-receptors are lacking. The dynamic interactions between the CRD, 7TM domain, and intracellular effectors are not fully characterized, limiting our understanding of their signaling mechanisms. Additionally, the influence of the extracellular matrix and lipid environment on receptor function is an emerging area that remains understudied. Recent advances in cryo-electron microscopy and molecular dynamics simulations offer promising tools to resolve the structural and functional intricacies of FZD receptors. These approaches could provide detailed insights into ligand binding, receptor activation, and the conformational changes underlying signal transduction. Novel chemical probes and antibody-based modulators targeting specific FZD structural domains are also being developed, with potential therapeutic applications for diseases driven by aberrant Wnt signaling. By addressing these controversies, unresolved issues, and research gaps, studying FZD receptor structures will enhance our understanding of their roles in cellular signaling and pave the way for innovative therapeutic strategies targeting Wnt-associated diseases.

## Role of FZD receptors in Wnt signaling

**FIGURE 2:**
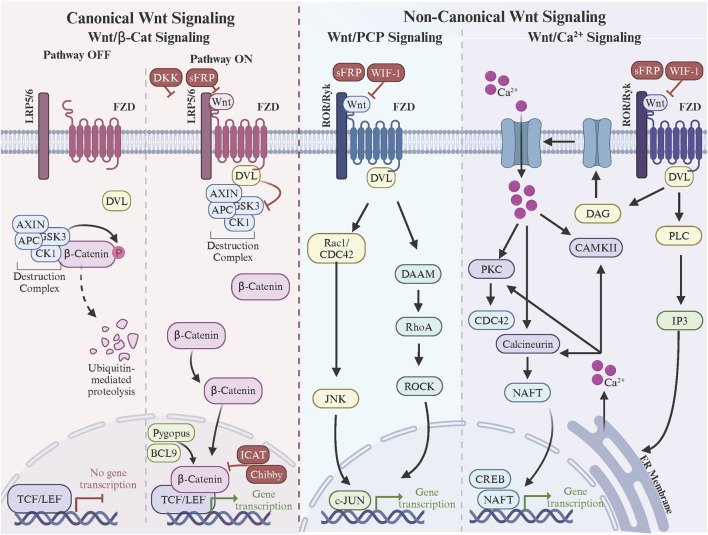
Frizzle regulation of canonical and non-canonical Wnt signaling. In canonical Wnt signaling, Wnt ligand binding to the FZD and LRP5/6 co-receptors triggers DVL binding to FZD C-terminal domain. DVL recruits the β-catenin destruction complex resulting in β-catenin stabilization, nuclear accumulation, and Wnt target gene expression via the TCF/LEF transcription factors. In non-canonical Wnt/PCP signaling, Wnt ligand binding to the FZD and ROR/Ryk co-receptors triggers the formation of FZD-PCP complexes (i.e., Vangl, Prickle, Cels, DVL). DVL leads activation of small Rho GTPases, cytoskeleton remodeling, and c-Jun-mediated gene transcription. Finally in non-canonical Wnt/Ca^2+^signaling, Wnt ligand binding to the FZD and ROR/Ryk co-receptors triggers the formation of FZD. Made with Biorender.com.

FZD receptors are indispensable mediators of the Wnt signaling pathways, which govern critical processes such as embryonic development, tissue homeostasis, and disease progression. These pathways are broadly categorized into the canonical Wnt/β-catenin and non-canonical pathways, including the Wnt/Ca^2+^ and PCP pathways (Figure 2). Each pathway utilizes specific FZD receptors to achieve distinct biological outcomes, but several controversies and unresolved questions persist regarding how these receptors achieve functional specificity and mediate diverse signaling mechanisms.

### Frizzled receptors and the canonical Wnt/β-catenin pathway

In the canonical Wnt/β-catenin signaling pathway, FZD receptors interact with Wnt ligands through their extracellular CRD and form functional complexes with co-receptors such as LRP5/6 (Hua et al., 2018). The CRD, approximately 120 amino acids in length, features 10 conserved cysteine residues that establish five disulfide bonds, stabilizing its fold and forming a hydrophobic groove crucial for ligand recognition (Chong et al., 2002b; Povelones and Nusse, 2005; Milhem and Ali, 2019). This groove interacts specifically with the lipid modifications of Wnt ligands, particularly the palmitoleic acid attached to Ser209 of Wnt3a, which inserts into the CRD’s hydrophobic pocket to anchor the ligand (Coombs et al., 2010). While this core interaction mechanism is shared among all FZD receptors, sequence variations in the CRD introduce receptor-specific affinities and specificities. For example, FZD7 and FZD10 exhibit strong interactions with canonical ligands like Wnt3a and Wnt1 (Cao et al., 2017; Alrefaei et al., 2020; Ding et al., 2020; Xu et al., 2020b), driven by their highly conserved hydrophobic pockets and additional stabilizing hydrogen bonds with Wnt peptides. In contrast, FZD5 demonstrates dual specificity, binding both canonical ligands like Wnt7a and non-canonical ligands like Wnt5a due to structural flexibility in its CRD (Tsutsumi et al., 2020; Li et al., 2022; Alsaadi et al., 2023).

The formation of a functional signaling complex also involves the co-receptors LRP5 and LRP6, which bind to a separate site on the Wnt ligand, distinct from the CRD-binding pocket (MacDonald and He, 2012). This bifurcated binding stabilizes the receptor-ligand interaction and facilitates the assembly of a ternary complex at the membrane (Tsutsumi et al., 2023). Structural studies have shown that the β-propeller and EGF-like domains of LRP5/6 interact with the Wnt ligand (Ahn et al., 2011), while their intracellular regions are phosphorylated by kinases like CK1 and GSK3 upon complex formation (Cselenyi et al., 2008; McCubrey et al., 2014). These phosphorylation events recruit Axin and disassemble the β-catenin destruction complex, allowing β-catenin to accumulate in the cytoplasm and translocate to the nucleus (Stamos and Weis, 2013). Additionally, FZD receptors are thought to form oligomers upon ligand binding, which, in combination with clustering of LRP5/6, enhances signal amplification by increasing the local concentration of signaling components (DeBruine et al., 2017; Hua et al., 2018). The transmembrane regions of FZD receptors and their interactions with co-receptors and the lipid bilayer further stabilize these higher-order complexes, ensuring efficient signal propagation (Xu et al., 2021b). Understanding these structural dynamics provides critical insights into how canonical Wnt signaling is mediated and regulated by the specific architecture of FZD receptors.

A critical mediator of this pathway is DVL, a cytoplasmic protein recruited to the intracellular C-terminal domain of FZD receptors upon ligand binding (Wallingford and Habas, 2005; Tauriello et al., 2012). DVL is a key signaling hub that integrates signals from FZD and transduces them downstream by inhibiting GSK3 activity within the destruction complex. DVL’s interaction with the PDZ-binding motif of the FZD C-terminal domain is essential for this process, and its phosphorylation state, regulated by kinases such as CK1, further modulates its activity (Wong et al., 2003; Bowin et al., 2023). DVL’s scaffolding function also aids in recruiting Axin to the membrane, sequestering it away from the β-catenin destruction complex (Li et al., 1999; Mallick et al., 2019). Downstream, stabilized β-catenin accumulates in the cytoplasm and translocates into the nucleus, where it interacts with the TCF/LEF (T-cell factor/lymphoid enhancer factor) family of transcription factors to activate Wnt target gene expression (Cadigan and Waterman, 2012). This nuclear activity is modulated by additional co-factors and chromatin remodelers, such as BCL9 and Pygopus, which enhance β-catenin’s transcriptional activity (Vafaizadeh et al., 2021). However, the nuclear translocation and activity of β-catenin are subject to tight regulation by inhibitors like ICAT (inhibitor of β-catenin and TCF) and Chibby, which disrupt β-catenin’s binding to TCF/LEF, thereby attenuating transcription (Tago et al., 2000; Ji et al., 2018).

Inhibitory mechanisms are also present upstream to ensure signaling specificity and prevent aberrant activation. Secreted inhibitors such as Dickkopf (DKK) and sFRPs (secreted Frizzled-related proteins) play a key role in modulating FZD receptor activity (Ahn et al., 2011). DKK binds to LRP5/6, preventing its interaction with FZD receptors and blocking canonical Wnt signaling (Cheng et al., 2011). sFRPs, on the other hand, bind directly to Wnt ligands or FZD CRDs, sequestering the ligands and reducing their availability to activate receptors (Chong et al., 2002a; Malinauskas et al., 2011; van Loon et al., 2021). The regulation of pathway inhibitors extends to intracellular mechanisms as well. Negative regulators like Axin are pivotal in reassembling the β-catenin destruction complex when Wnt signaling subsides (Ranes et al., 2021). Phosphatases, such as PP2A, also play a role in resetting the pathway by dephosphorylating key components, including LRP5/6 and DVL, thereby restoring their inactive states (Townsley, 1999; Yamamoto et al., 2001). Additionally, ubiquitin ligases such as ZNRF3 and RNF43 inhibit Wnt signaling by ubiquitinating FZD receptors, marking them for internalization and degradation (Zebisch et al., 2013; Farnhammer et al., 2023).

The delicate balance between activators and inhibitors ensures that canonical Wnt signaling is precisely controlled in both spatial and temporal dimensions. Dysregulation of these downstream components, whether through overactivation of β-catenin or loss of inhibitory control, is a hallmark of many diseases, including cancers and developmental disorders. Some studies emphasize the role of the extracellular CRD in ligand binding, while others highlight the importance of co-receptor interactions, receptor clustering, and cellular context. Moreover, the relative contributions of FZD receptors versus co-receptors like LRP5/6 in canonical signaling fidelity remain a key area of debate. Understanding the molecular details of these interactions provides crucial insights into how FZD receptors mediate signal transduction and how the pathway can be therapeutically targeted.

### Frizzled receptors in non-canonical Wnt signaling

Unlike canonical Wnt/β-catenin signaling, the Wnt/Ca^2+^ and PCP pathways function independently of β-catenin stabilization, activating distinct downstream effectors that remodel the cytoskeleton, enhance cell motility, and alter tumor microenvironments (De, 2011; Gao, 2012). In certain cancers, such as gastric cancer and melanoma, noncanonical signaling of FZD7 can also contribute to cancer phenotypes (Li et al., 2018; Rodriguez-Hernandez et al., 2020). The structural versatility of FZD receptors, particularly FZD3, FZD5, and FZD6, enables their interaction with non-canonical Wnt ligands such as Wnt5a and Wnt11, which are often overexpressed in cancers, including breast, colorectal, and melanoma (Corda and Sala, 2017; Brandt et al., 2018; Li C. et al., 2019).

The Wnt/Ca^2+^ pathway is initiated when Wnt5a or Wnt11 binds to the CRD of FZD receptors, inducing conformational changes in the 7TM domain (Doi et al., 2010; Sarabia-Sánchez et al., 2023). This activates heterotrimeric G-proteins, which stimulate phospholipase C (PLC) to hydrolyze phosphatidylinositol 4,5-bisphosphate (PIP2) into inositol trisphosphate (IP3) and diacylglycerol (DAG) (Zhang et al., 2020). The release of intracellular Ca^2+^ stores, triggered by IP3, and the activation of protein kinase C (PKC) by DAG drive critical downstream events, including the activation of Ca^2+^/calmodulin-dependent protein kinase II (CaMKII) and calcineurin (Kühl et al., 2000). These effectors influence transcriptional regulators such as NFAT (nuclear factor of activated T-cells) and CREB, modulating gene expression programs that promote tumor cell survival, migration, and angiogenesis (Gregory et al., 2010). Elevated Wnt/Ca^2+^ signaling has been observed in various cancers, correlating with increased metastasis. For instance, Wnt5a-FZD interactions enhance Ca^2+^-mediated cytoskeletal reorganization and cell motility, facilitating tumor invasion into surrounding tissues and dissemination to distant sites.

The PCP pathway, mediated by FZD3 and FZD6, further amplifies non-canonical Wnt signaling’s role in cancer progression (Dong et al., 2018). In this pathway, Wnt5a or Wnt11 binds the CRD of FZD receptors, forming asymmetric signaling complexes with core PCP proteins such as Vangl, Prickle, Celsr, and DVL (Dreyer et al., 2022; Seo et al., 2023). The recruitment of DVL to the intracellular C-terminal domain of FZD triggers the activation of small Rho family GTPases, including RhoA, Rac1, and Cdc42. These GTPases orchestrate cytoskeletal remodeling, driving the formation of lamellipodia and filopodia, which are crucial for directional migration and invasive behavior (Mezzacappa et al., 2012). Rac1 further activates c-Jun N-terminal kinase (JNK), a key effector that regulates transcription and cytoskeletal dynamics. In triple-negative breast and melanoma, PCP signaling enhances collective and single-cell migration, contributing to metastatic potential (Yamanaka et al., 2002). Moreover, PCP signaling influences tumor microenvironment remodeling by altering the orientation and behavior of stromal cells, including fibroblasts and endothelial cells, which support tumor growth and angiogenesis (VanderVorst et al., 2019; Rogers et al., 2023).

The Wnt/Ca^2+^ and PCP pathways are tightly regulated by activators and inhibitors that modulate FZD receptor activity. Key activators include PLC, CaMKII, and Rho GTPases, which propagate downstream signals, while secreted inhibitors such as sFRPs and WIF-1 sequester Wnt ligands, limiting their interaction with FZD receptors. E3 ubiquitin ligases like ZNRF3 and RNF43 also ubiquitinate FZD receptors, promoting their degradation and reducing signaling output. However, in cancer, these regulatory mechanisms are frequently disrupted. Overexpression of Wnt5a or mutations in FZD receptors can lead to constitutive activation of non-canonical signaling, bypassing normal regulatory controls. For example, FZD6 is upregulated in several cancers, where its enhanced PCP signaling promotes epithelial-to-mesenchymal transition (EMT), a critical step in metastasis. Similarly, dysregulated Wnt/Ca^2+^ signaling has been implicated in therapy resistance, as Ca^2+^-mediated signaling can activate pro-survival pathways that counteract apoptosis.

Critical gaps remain in understanding how non-canonical Wnt signaling contributes to cancer heterogeneity and therapy resistance. The interplay between canonical and non-canonical pathways is particularly complex, as ligands like Wnt5a can activate both, with context-dependent effects on tumor progression (Yuzugullu et al., 2009). The dynamic regulation of FZD signaling remains a fundamental challenge in the field. Understanding how FZD receptors integrate signals across Wnt pathways, adapt to cellular contexts, and achieve specificity in downstream signaling is critical for addressing their roles in development and disease.

## Role of FZD receptors in cell crosstalk

Frizzled receptors not only transmit signals from lipid-modified Wnt ligands but also integrate physical cues from the extracellular matrix (ECM), allowing cells to respond dynamically to both their neighbors and their mechanical environment (Du et al., 2016). In breast cancer, for example, cancer-associated fibroblasts (CAFs) are major producers of non-canonical Wnt ligands like Wnt5a (Hu D. et al., 2022). These ligands bind to FZD5 and FZD6 receptors on adjacent tumor epithelial cells, activating pathways that promote epithelial-to-mesenchymal transition, motility, and resistance to chemotherapy (Xu et al., 2020a). This crosstalk is intensified by ECM stiffening, which enhances clustering of FZD receptors at the plasma membrane, increasing downstream signaling through pathways such as PCP and calcium signaling (Asif et al., 2021). The interaction between CAFs and tumor cells via FZD receptors forms a critical axis in the tumor microenvironment that supports invasion and metastasis (Wu et al., 2021). Furthermore, crosstalk between the Wnt and transforming growth factor β1 (TGFβ) pathways exacerbates fibrosis in Crohns disease (Lewis et al., 2022). In this disease model, TGFβ upregulated the expression of Wnt5b, FZD8, and collagen-I, a major ECM component. Inhibiting non-canonical Wnt signaling through the FZD8 specific inhibitor, 3235-0367, decreased fibroblast’s expression of various ECM components upon TGFβ stimulation. These findings underscore how FZD receptors play a key role in ECM composition that can be therapeutically targeted.

In the skeletal system, FZD-mediated crosstalk maintains the delicate balance between bone formation and resorption (Hu et al., 2024). Osteoblasts secrete Wnt ligands, particularly Wnt10b and Wnt16, which signal through FZD9 on osteoclast precursors, inhibiting their differentiation and thus limiting bone resorption (David, 2011). This paracrine signaling helps maintain bone density and structural integrity. Disruption of this pathway, as seen in aging or inflammatory conditions, contributes to osteoporosis by tilting the balance toward excessive osteoclast activity. Similarly, in the fibrotic liver, damaged hepatocytes release canonical Wnt ligands like Wnt3a and Wnt7a, which engage FZD2 and FZD8 on hepatic stellate cells (HSCs) (Rutt et al., 2024). These interactions induce a myofibroblastic phenotype in HSCs, leading to increased secretion of ECM components such as collagen I and fibronectin. The accumulating matrix further stiffens the hepatic environment, reinforcing FZD receptor signaling in a profibrotic feedback loop that progressively impairs liver function. In a similar fashion, crosstalk between Wnt2b secreting fibroblasts and natural killer (NK) cells contributes to fibrosis in inflammatory bowel disease (IBD) (Cheng et al., 2024). Cheng and colleges observed that Wnt2b secreted by fibroblasts activates FZD4 on NK cells and triggers IL-33 expression via NF- κ B and STAT3 pathways. This was achieved by Wnt2b-FZD4-mediated activation of TCF4, the canonical Wnt-regulated transcription factor, which bound to p65 and enhanced IL-33 promoter binding. Inhibiting this pathway pharmacologically, using Salinomycin, or genetically knocking down WNT2b expression in fibroblasts reduced colonic inflammation and fibrosis.

FZD receptors also play a vital role in tumor–endothelial cell crosstalk during metastasis. Endothelial cells in pre-metastatic niches can secrete Wnt ligands that interact with FZD7 on circulating tumor cells, enhancing their survival, adhesion, and outgrowth in distant tissues (Zhang and Xu, 2022). Conversely, tumor cells can influence endothelial behavior by releasing Wnts that activate FZD-mediated signaling in endothelial cells, promoting angiogenesis and vascular permeability—conditions favorable for tumor expansion and dissemination (Dejana, 2010; Olsen et al., 2017). In the central nervous system, the interaction between microglia and neural progenitor cells is another context in which FZD signaling regulates cell fate decisions (Knotek et al., 2020). During neuroinflammatory events, such as those seen in multiple sclerosis, activated microglia produce Wnt1, which binds FZD1 on neural progenitors (Tawk et al., 2011). This modulates neural stem cell proliferation and differentiation, with the potential to either support repair or, under chronic stress, contribute to maladaptive neurogenesis or demyelination.

Altogether, these examples illustrate the centrality of FZD receptors in mediating cell-cell communication in both regenerative and pathological contexts. They serve as critical sensors and transducers of extracellular cues—both molecular and mechanical—shaping how cells interact with their environment and with each other. Disruption or hyperactivation of these FZD-mediated crosstalk pathways is a common feature of diverse diseases, making them attractive targets for therapeutic intervention across oncology, fibrosis, neurology, and regenerative medicine.

## Regulation of FZD receptors

**FIGURE 3:**
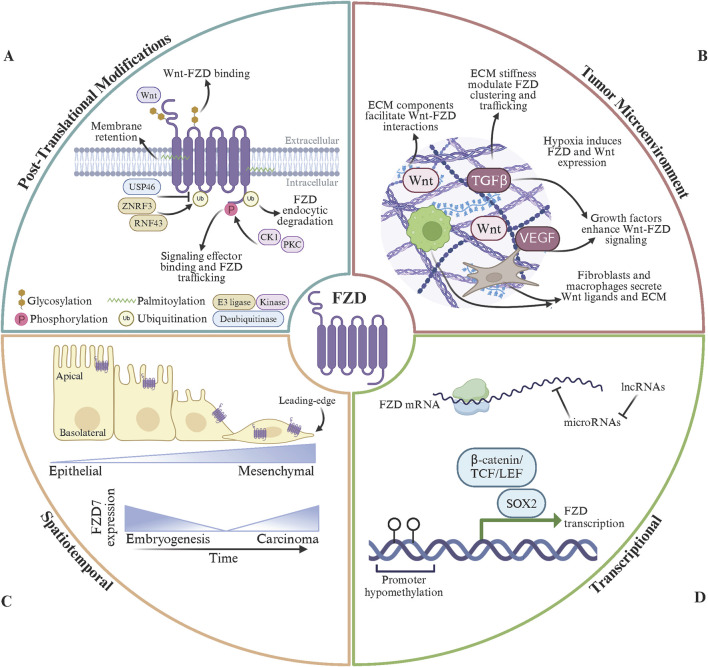
Modes of FZD receptor regulation. **(A)** FZD post-translational modifications (PTMs) include glycosylation, phosphorylation, palmitoylation, and ubiquitination. N-linked glycosylation in FZD extracellular domains stabilizes Wnt binding and facilitates FZD protein folding. Phosphorylation of the FZD C-terminal domain, by CK1 or PKC, regulate interactions between FZD and intracellular signal effector proteins and FZD trafficking dynamics. Palmitoylation of FZD intracellular regions promotes membrane retention and sustained signaling at the cell surface. FZD endocytosis, degradation, and subsequent signal termination is regulated by ubiquitin PTMs per the dynamic activity of E3 ubiquitin ligases (ZNRF3 and RNF43) and deubiquitinating enzymes (USP46). **(B)** The tumor microenvironment (TME) contributes to Wnt ligand bioavailabiliy, FZD receptor localization, and downstream receptor signaling. Extra-cellular matrix (ECM) components bind to Wnt to facilitating ligand presentation and stabilize Wnt-FZD interactions. ECM stiffness regulates FZD receptor clustering and intracellular trafficking to enhance Wnt signaling. The hypoxic tumor microenvironment can stimulate FZD receptor and Wnt ligand overexpression. Growth factors present in the TME synergistically enhance Wnt/FZD signaling to promote tumor growth and metastasis. Fibroblasts and macrophages residing within the TME can secrete Wnt ligands and ECM components to further propagate FZD signaling. **(C)** Spatiotemporal regulation of FZD is essential for proper FZD expression during tissue development, cell polarity, and signaling dynamics. FZD receptor expression is tightly controlled during development and results in temporal and tissue-specific expression patterns. Within epithelial cells, FZD receptors are located to the apical membrane and mediate planar cell polarity. Aberrant FZD receptor expression and mislocalization of FZD receptors disrupts normal signaling pathways during tumorigenesis. **(D)** FZD gene transcriptional products are regulated at the genomic and epigenetic levels and are susceptible to alterations in cancer. Developmental transcription factors (SOX2, OCT4, NANOG), the β-catenin/TCF/LEF complex, and HIF1α activate FZD gene transcription. Epigenetically, FZD mRNA abundance is regulated by promoter methylation status, histone acetylation levels, and functional chromatin remodeler complexes. In addition, microRNAs (miR-126 and miR-200) target FZD mRNA for degradation and lncRNAs (MALAT1 and HOTAIR) sponge microRNAs to enhance FZD mRNA availability. Finally, FZD receptors undergo gene amplification (FZD7/10) and loss-of-function mutations (FZD9) in cancer. Made with Biorender.com.

The regulation of FZD receptors is a dynamic and multifaceted process that controls their signaling activity and ensures appropriate cellular responses to Wnt signals. Key regulatory mechanisms include post-translational modifications (PTMs) (Pascual-Vargas and Salinas, 2021), spatial organization, receptor endocytosis and recycling (Brunt and Scholpp, 2018), interactions with co-receptors (Ko et al., 2022), inhibitors (Gurney et al., 2012), and ECM (Li J. et al., 2024), and gene regulation (Ueno et al., 2013; Alrefaei, 2021) (Figure 3). These mechanisms operate in concert to fine-tune the strength, duration, and specificity of FZD-mediated signaling. However, unresolved questions and differing perspectives persist regarding these regulatory processes’ precise molecular details and broader implications.

### Post-translational modifications

PTMs play a pivotal role in regulating the activity, stability, and trafficking of FZD receptors, ensuring proper control of Wnt signaling. Phosphorylation is one of the most well-studied PTMs, occurring primarily on the intracellular C-terminal domain of FZD receptors (Strakova et al., 2018). Kinases such as casein kinase 1 (CK1) and protein kinase C (PKC) phosphorylate FZD receptors to modulate their interactions with signaling proteins and their trafficking dynamics. For example, CK1 phosphorylates conserved serine and threonine residues on FZD6, promoting its interaction with DVL to activate the PCP pathway (Bernatík et al., 2014). In cancers such as melanoma, dysregulated CK1-mediated phosphorylation of FZD6 enhances PCP signaling, driving EMT and metastasis (Dong et al., 2023). Similarly, PKC, activated downstream of the Wnt/Ca^2+^ pathway, phosphorylates FZD3 and FZD5, facilitating their recycling to the plasma membrane (Bengochea et al., 2008). This recycling sustains non-canonical signaling in highly invasive cancers like triple-negative breast cancer, where prolonged Wnt/Ca^2+^ activity promotes tumor cell migration and invasion (Pohl et al., 2017). Conversely, hyperphosphorylation of FZD7 in the absence of Wnt ligands marks the receptor for degradation, providing a mechanism to attenuate canonical Wnt signaling (Yang et al., 2011; Fernandez et al., 2014). In colorectal cancers, mutations in kinases or phosphatases that regulate this phosphorylation can lead to aberrant stabilization of FZD7, contributing to excessive Wnt/β-catenin activity and tumor proliferation (Ueno et al., 2008).

Ubiquitination is another critical PTM that governs the endocytosis and degradation of FZD receptors (Stevens and Williams, 2021). E3 ubiquitin ligases such as ZNRF3 and RNF43 ubiquitinate FZD receptors, targeting them for lysosomal degradation and as a negative feedback mechanism to terminate Wnt signaling (Jiang et al., 2015; Farnhammer et al., 2023). Mutations or loss of function in ZNRF3 or RNF43 are frequently observed in cancers such as colorectal, pancreatic, and ovarian cancers, resulting in FZD receptor overexpression and persistent Wnt signaling (Jiang et al., 2013; Ryland et al., 2013; Giannakis et al., 2014; Yamamoto et al., 2022). These mutations are often associated with increased tumor growth, therapy resistance, and poor prognosis. Additionally, deubiquitinating enzymes, such as USP46, have been shown to stabilize FZD receptors by reversing ubiquitination, further enhancing signaling in cancerous cells (Cho et al., 2022). In the tumor microenvironment, these alterations in ubiquitination pathways amplify oncogenic signaling and contribute to cancer cell plasticity and metastatic potential.

Other PTMs, including palmitoylation and glycosylation, influence FZD receptor function and localization. Palmitoylation of intracellular cysteine residues can enhance the membrane retention of FZD receptors, supporting sustained signaling driving tumorigenesis (Zheng et al., 2022). Glycosylation, particularly N-linked glycosylation in the extracellular domains, stabilizes receptor-ligand interactions and facilitates proper folding of the FZD protein (Bhanot et al., 1996; Schulte and Bryja, 2007). Alterations in glycosylation patterns in cancer cells have been shown to enhance the binding affinity of FZD receptors for Wnt ligands, further exacerbating dysregulated signaling (Guo et al., 2014). Together, these PTMs tightly regulate FZD receptor activity under normal physiological conditions, but their dysregulation in cancer creates an environment of hyperactive Wnt signaling. This dysregulation promotes tumor growth, invasion, and resistance to therapies (Barkeer et al., 2018). Targeting the enzymes responsible for these PTMs, such as specific kinases, E3 ligases, or deubiquitinating enzymes, represents a promising therapeutic avenue for cancers driven by aberrant FZD signaling. Future research is needed to understand the interplay of these modifications better and develop precision-targeted therapies to restore normal FZD regulation.

### Spatial organization

The regulation of FZD receptor expression and localization is critical for their function in tissue development, cellular polarity, and signaling dynamics. In cancer, aberrant expression and mislocalization of FZD receptors disrupt normal signaling pathways, driving tumorigenesis, invasion, and metastasis (Mukai et al., 2010). Each FZD receptor exhibits tissue-specific expression patterns, which are tightly controlled during development but become dysregulated in many cancers (Yang et al., 2024). For instance, FZD7 is highly expressed in the intestinal epithelium and embryonic stem cells, where it maintains stemness and promotes proliferation through canonical Wnt signaling (Flanagan et al., 2015). However, in colorectal and gastric cancers, FZD7 is overexpressed, amplifying β-catenin signaling to sustain tumor growth and resistance to therapy (Phesse et al., 2016; Li et al., 2018; Ye et al., 2019). Similarly, FZD10, minimally expressed in most adult tissues, is overexpressed in synovial sarcoma and hepatocellular carcinoma, driving tumor progression via enhanced canonical Wnt signaling (Nagayama et al., 2005; Chan and Lo, 2018). These examples underscore how tissue-specific FZD expression becomes co-opted in cancer to promote malignant behaviors.

The temporal and spatial regulation of FZD receptors during development is also subject to dysregulation in cancer. In normal development, FZD receptors are expressed at specific times to coordinate processes like gastrulation, organogenesis, and neural tube closure (Chuan et al., 2024). For example, FZD5 and FZD7 are expressed early in embryogenesis to regulate stem cell proliferation and differentiation (Kemp et al., 2007). However, these receptors are reactivated or overexpressed in cancer, mimicking developmental programs to sustain tumor growth (Thiele et al., 2018; Sun et al., 2020; Xia et al., 2024). The spatial organization of FZD receptor trafficking also contributes to cancer progression. In polarized epithelial cells, FZD receptors are typically restricted to specific membrane domains, such as the apical or basolateral surface, ensuring localized Wnt signaling (Strakova et al., 2018; Ghimire S. and Deans M., 2019; Ghimire S. R. and Deans M. R., 2019). This polarity is often lost in cancer cells, and FZD receptors are redistributed to the leading edge of migrating cells or to intracellular signaling endosomes (Peglion and Etienne-Manneville, 2024). This mislocalization amplifies signaling pathways, such as the PCP and Wnt/Ca^2+^ pathways, enabling directional migration, invasion, and metastasis.

Endocytosis and recycling of FZD receptors are critical processes that regulate their availability on the cell surface and control the strength, duration, and spatial organization of Wnt signaling. These trafficking mechanisms ensure that FZD receptors are internalized to amplify signaling within endosomes or attenuate signaling through lysosomal degradation (Pascual-Vargas and Salinas, 2021; Zheng et al., 2022). Dysregulation of these processes is commonly observed in cancer, where altered FZD trafficking enhances oncogenic signaling and contributes to tumor growth, invasion, and metastasis. Endocytosis of FZD receptors is initiated after ligand binding, with both clathrin-dependent and clathrin-independent mechanisms implicated (Kirchhausen et al., 2014; Brunt and Scholpp, 2018; Agajanian et al., 2019; Nguyen et al., 2024). Canonical Wnt ligands, such as Wnt3a, promote the internalization of FZD receptors into early endosomes, where signaling components, including DVL and Axin, assemble to sustain β-catenin stabilization (Zheng and Sheng, 2024). This endosomal signaling compartment is crucial for amplifying canonical Wnt/β-catenin signaling (Colozza and Koo, 2021). Conversely, in the absence of Wnt ligands, FZD receptors are internalized and targeted for lysosomal degradation to terminate signaling. Ubiquitination by E3 ligases, such as ZNRF3 and RNF43, marks FZD receptors for degradation (Jiang et al., 2015). Mutations or loss of function in ZNRF3 and RNF43 prevent proper receptor degradation, leading to sustained FZD receptor presence on the cell surface, hyperactivation of Wnt signaling, and enhanced tumor cell proliferation (Li S. et al., 2024). Recycling of FZD receptors back to the plasma membrane is another critical process that allows cells to remain responsive to Wnt ligands (Hao et al., 2012; Koo et al., 2012). Rab GTPases, such as Rab8, regulate endosomal recycling, which direct FZD receptors to recycling endosomes for transport back to the plasma membrane (Stypulkowski et al., 2021). Dysregulation of this process in cancer cells enhances non-canonical signaling, promoting invasive and migratory behaviors. Cancer cells often exploit endocytic trafficking to shift the balance between receptor recycling and degradation in favor of sustained signaling (Khan and Steeg, 2021).

Advanced imaging technologies have transformed FZD receptor research by enabling real-time, high-resolution visualization of receptor dynamics in living cells and tissues. Super-resolution microscopy techniques, such as stochastic optical reconstruction microscopy (STORM) and stimulated emission depletion (STED) microscopy, have made it possible to observe FZD receptor clustering, trafficking, and interactions with signaling partners like DVL at nanoscale resolution (Sengupta et al., 2014; Mii et al., 2017; Ban et al., 2021; Schubert et al., 2022). Single-molecule tracking has been particularly useful for understanding the spatiotemporal dynamics of FZD receptors, revealing how they are internalized, recycled, or degraded in response to Wnt ligand stimulation. These imaging tools have clarified how receptor localization and trafficking regulate Wnt signaling intensity and duration, with implications for developmental processes and tumorigenesis. For example, studies using live-cell imaging have shown that aberrant recycling of FZD receptors, such as FZD7, sustains hyperactive Wnt signaling in colorectal cancer, driving tumor proliferation and resistance to therapy.

### Extracellular matrix

The ECM and the broader cellular environment play critical roles in modulating FZD receptor signaling, significantly influencing their function in both physiological and pathological contexts. The ECM, composed of proteins such as collagen, fibronectin, laminins, and proteoglycans, serves as a dynamic scaffold that regulates cell adhesion, migration, and signaling. For FZD receptors, the ECM acts as a physical substrate and a biochemical modulator, shaping the availability of Wnt ligands, receptor localization, and downstream signaling (Bobbs et al., 2015; Li J. et al., 2024). In cancer, alterations in the ECM and tumor microenvironment (TME) often co-opt these processes to promote tumor progression, invasion, and metastasis (Sompel et al., 2021).

ECM components, such as heparan sulfate proteoglycans (HSPGs), directly regulate FZD receptor signaling by facilitating Wnt ligand presentation (O'Connell et al., 2009; Li N. et al., 2019). HSPGs like glypicans and syndecans bind to Wnt ligands and stabilize their interactions with the CRD of FZD receptors, enhancing the efficiency of Wnt signaling. For instance, glypican-3 (GPC3) enhances Wnt/β-catenin signaling by concentrating Wnt ligands near FZD receptors, a mechanism frequently observed in hepatocellular carcinoma (Kolluri and Ho, 2019; Li N. et al., 2019). Similarly, syndecans interact with Wnt5a to regulate Wnt signaling pathways, including those mediated by FZD3 and FZD6, which are critical for processes like cell migration and polarity (O'Connell et al., 2009; Ren et al., 2018). Dysregulation of these ECM components in the TME often amplifies FZD-mediated Wnt signaling, supporting tumor cell proliferation, invasion, and resistance to apoptosis.

The physical properties of the ECM, such as stiffness and tension, also profoundly influence FZD receptor signaling (Poltavets et al., 2018). The stiffness of the ECM plays a pivotal role in the development and progression of various diseases, particularly cancer, fibrosis, and cardiovascular disorders (Handorf et al., 2015; Mancini et al., 2024). This is in part due to increased deposition of matrix proteins such as collagen, enhanced cross-linking by enzymes like lysyl oxidase (LOX), and sustained inflammation (Vallet and Ricard-Blum, 2019; Pfisterer et al., 2021; Park et al., 2024). In tissues undergoing tumorigenesis, increased ECM stiffness, often due to excessive collagen deposition and crosslinking, alters the spatial organization of FZD receptors and their associated signaling complexes (Wullkopf et al., 2018; Deng et al., 2022; Mai et al., 2024). For example, ECM stiffening promotes clustering of FZD7 and LRP6 on the cell surface, enhancing canonical Wnt/β-catenin signaling in colorectal and breast cancers (King et al., 2012; Zhou et al., 2019; Fico and Santamaria-Martínez, 2020; Yin et al., 2020). In fibrotic tissues, sustained mechanical stress reinforces Wnt/FZD signaling loops that perpetuate fibroblast activation and matrix overproduction (Somanader et al., 2024). Cardiovascular tissues also exhibit this interplay, as stiffened ECM contributes to endothelial dysfunction partly through altered FZD-mediated signaling pathways that regulate vascular remodeling and inflammation (Kurose, 2021). Similarly, mechanical forces transmitted through integrins and cytoskeletal components can modulate the trafficking and recycling of FZD receptors, sustaining non-canonical pathways like Wnt/PCP that drive tumor cell migration and invasion. Thus, the integration of ECM stiffness and FZD activity forms a critical axis in disease progression, making both attractive targets for therapeutic intervention. Agents that modulate matrix stiffness or disrupt aberrant FZD signaling hold promise for reversing pathological tissue remodeling and restoring homeostasis.

Crosstalk between the ECM, growth factors, and FZD receptors is another critical aspect of their regulation in cancer. Growth factors such as transforming growth factor-beta (TGF-β) often work synergistically with Wnt/FZD signaling to promote tumor growth and metastasis (Chen et al., 2016; Spanjer et al., 2016). For instance, TGF-β signaling enhances the expression of ECM components like fibronectin, which binds to syndecans and facilitates FZD-mediated Wnt signaling (Murillo-Garzón et al., 2018). This interaction is particularly evident in cancers undergoing EMT, where the ECM remodels to support invasive and migratory tumor phenotypes (Li et al., 2018; Tuluhong et al., 2021). In addition to promoting tumor growth and metastasis, the ECM and cellular environment influence FZD receptor signaling in stromal and immune cells, shaping the TME. Fibroblasts within the ECM can secrete Wnt ligands, such as Wnt2, to activate FZD receptors on neighboring tumor cells, enhancing invasion and immune evasion (Aizawa et al., 2019). Similarly, tumor-associated macrophages (TAMs) can secrete Wnt5a to act as cellular sources of Wnt ligands for FZD signaling (Kim et al., 2012; Valencia et al., 2014). Different DVL paralogs have been implicated in the regulation of immunomodulatory genes (Castro-Piedras et al., 2021; Rasha et al., 2023), but which FZD-DVL networks mediate this downstream signaling remains poorly understood (Sharma and Pruitt, 2020).

The dysregulation of ECM-FZD interactions in cancer highlights the potential for therapeutic intervention. Strategies targeting the ECM, such as inhibitors of collagen crosslinking enzymes or HSPG-binding molecules, could disrupt FZD-mediated signaling and reduce tumor progression. Additionally, therapies to restore ECM stiffness or remodel tumor-associated ECM could normalize FZD receptor localization and attenuate aberrant Wnt signaling. Further research into the dynamic interplay between the ECM, FZD receptors, and the TME is essential to understand their contributions to cancer progression fully and to develop precision therapies targeting these critical interactions.

### Transcriptomic and genomic regulation

The regulation of FZD receptors at the transcriptomic and genomic levels is essential for ensuring proper Wnt signaling during development, tissue homeostasis, and repair. This regulation involves transcription factors, enhancers, epigenetic modifications, and non-coding RNAs, which collectively control the spatial and temporal expression of FZD receptors. Dysregulation of these mechanisms is frequently observed in cancer, where altered FZD expression contributes to tumor growth, invasion, and metastasis. Transcriptional control is largely governed by developmental transcription factors, such as SOX2, OCT4, and NANOG, which regulate the expression of FZD7 in embryonic stem cells (Melchior et al., 2008; Fernandez et al., 2014; Meng et al., 2020; Mukherjee et al., 2022). These factors are often reactivated in cancers like glioblastoma and gastric cancer, driving FZD overexpression and promoting tumor stemness. Additionally, the β-catenin/TCF/LEF complex, a hallmark of canonical Wnt signaling, regulates FZD transcription as part of a positive feedback loop (Hrckulak et al., 2016). Analysis of a genomic region 7 kb upstream of the transcription start site of the *FZD7* gene revealed that c-Jun binds to multiple upstream regions. Interestingly, 2 kb upstream of the TSS of the FZD7, DVL1 and β-catenin were shown to bind. The binding of these factors were sensitive to sirtuin activity as the inhibition of SIRT1 led to a reduction in the occupancy of these factors in breast cancer models (Simmons et al., 2014).

Epigenetic regulation modulates FZD expression through DNA methylation, histone modifications, and chromatin remodeling (Zhu and Zhou, 2024). Hypermethylation of FZD promoter regions is associated with silencing in certain cancers, as seen with FZD9 in leukemia, leading to impaired Wnt signaling (Zhang et al., 2016). Conversely, hypomethylation of FZD promoters is a common feature in colorectal and liver cancers, resulting in their overexpression and sustained oncogenic signaling (Galamb et al., 2016; Palomer et al., 2022; Wang et al., 2022; Yang et al., 2023). Histone acetylation and methylation also influence chromatin accessibility at FZD loci, with histone acetyltransferases (HATs) enhancing FZD expression in tumors by loosening chromatin structure (Deng et al., 2024; Wang et al., 2014). Non-coding RNAs, including microRNAs (miRNAs) and long non-coding RNAs (lncRNAs), add an additional layer of FZD post-transcriptional regulation (Ueno et al., 2013). Multiple miRNAs impact FZD receptors in both solid and hematologic malignancies, possessing the ability to modulate various branches of Wnt signaling (Salvi et al., 2009; Tong et al., 2009; White et al., 2011; Krützfeldt et al., 2012; Jiang et al., 2016; Kenneth et al., 2023). LncRNAs such as MALAT1, HOTAIR, and MAFG-DT, act as sponges for miRNAs that target FZD mRNAs, indirectly upregulating FZD receptors (Hakami et al., 2024; Wang et al., 2024; Wu et al., 2024). In the case of prostate cancer, MAFG-DT is overexpressed and sequesters miR-24-3p to upregulate FZD4 and FZD5 mRNA, enhance Wnt/β-catenin signaling and ultimately bone metastasis (Wang et al., 2024). This mechanism is particularly prominent in breast and colorectal cancers, where lncRNA-mediated FZD overexpression is associated with enhanced tumor growth and poor prognosis (Smith et al., 2021; Lu et al., 2023).

FZD receptors are subject to alterations such as amplifications, mutations, and deletions at the genomic level (Zeng C.-M. et al., 2018; Li et al., 2021; Sun et al., 2021; Hafezi et al., 2022a). Mutations in FZD genes, particularly in the extracellular CRD, can disrupt ligand binding or create constitutively active receptors, as observed in some aggressive cancers. Loss-of-function mutations, such as those affecting FZD9, are linked to conditions like leukemia, where reduced Wnt signaling impairs normal cellular processes (Sompel et al., 2021). Functional genomics has also made significant contributions to the field. CRISPR-Cas9 gene editing has been a powerful tool for studying the roles of individual FZD receptors in development and disease (Voloshanenko et al., 2017; Kerek et al., 2020). Knockout and knockdown experiments have revealed context-specific functions of FZD receptors, such as the role of FZD4 in angiogenesis and FZD6 in planar cell polarity (Steinhart et al., 2017; Han et al., 2024). Single-cell and spatial transcriptomics have provided detailed maps of FZD receptor expression across different tissues, developmental stages, and disease states (Hu S. et al., 2022; Gayden et al., 2023). These approaches have identified unique expression profiles of FZD receptors in tumor microenvironments, highlighting their potential as diagnostic markers and therapeutic targets. Multiple FZD receptors show increased expression in cancer lines (Simmons et al., 2014; Castro-Piedras et al., 2021), and some FZDs, such as FZD7, show a decrease in mRNA and protein expression in response to sirtuin inhibitors (Castro-Piedras et al., 2021; Simmons et al., 2014).

Despite substantial progress, several research gaps remain. The temporal dynamics of receptor endocytosis, recycling, and degradation are poorly understood, as are the mechanisms by which these processes differ across cell types and signaling contexts. Additionally, the interplay between mechanical cues from the ECM and FZD signaling is an emerging area that requires further exploration. These advancements deepen our understanding of the structural and functional diversity of FZD receptors and drive therapeutic innovation. Integrating structural, imaging, and functional data enables the development of selective therapeutics targeting FZD-driven pathways in cancer, fibrosis, and degenerative diseases. Continued progress in these technologies promises to unlock new insights into FZD receptor biology and translate these findings into clinical applications.

## FZD as therapeutic targets

FZD receptors have emerged as attractive therapeutic targets due to their critical roles in regulating Wnt signaling, which is often dysregulated in cancer (Zeng C. M. et al., 2018). Aberrant FZD signaling contributes to tumor initiation, progression, metastasis, and therapy resistance, making these receptors key intervention points in oncology. Therapeutic approaches targeting FZD receptors include monoclonal antibodies, small-molecule inhibitors, decoy receptors, RNA-based therapies, and emerging gene-editing technologies (Nagayama et al., 2005; Lee et al., 2015; Pavlovic et al., 2018; Kozielewicz et al., 2020; Hafezi et al., 2022b; Svilenov, 2023; Han et al., 2024) (Table 1). Among the most prominent efforts, monoclonal antibodies like OMP-18R5 (Vantictumab) target multiple FZD receptors (e.g., FZD1, FZD2, FZD5, FZD7, and FZD8), blocking both canonical and non-canonical Wnt signaling. Vantictumab has shown efficacy in reducing tumor growth and cancer stem cell populations in preclinical colorectal, pancreatic, and breast cancer models (Gurney et al., 2012; Davis et al., 2020; Diamond et al., 2020). However, clinical trials revealed dose-limiting toxicities, such as bone fragility, highlighting the challenge of targeting a pathway critical for normal tissue homeostasis. More selective antibodies and other therapeutics, such as those targeting FZD7, are being developed to inhibit canonical Wnt signaling specifically in cancers like colorectal and gastric tumors, where FZD7 overexpression drives tumor growth and resistance to apoptosis (Hua et al., 2022; Larasati et al., 2022). Interestingly, one preclinical study demonstrated lack of efficacy for FZD7 inhibition of colorectal tumors containing APC or β-catenin mutations (Gurney et al., 2012). Mutated APC is found in >80% of colorectal cancers, indicating that colorectal cancers may require an alternative therapy (Parker and Neufeld, 2020).

**TABLE 1:** Summary of FZD cancer therapeutics and approaches in preclinical models. To perturb downstream Wnt-FZD signaling and cancer progression, therapeutics have been developed to reduce FZD mRNA expression, inhibit FZD conformational changes upon Wnt binding, antagonize endogenous FZD for Wnt binding, vaccinate against FZD members, and block upstream Wnt secretion.

Therapy	Therapeutic Agent	Target	Effect	Citation
Monoclonal antibodies	OMP-18R5 (Vantictumab)	FZD1, FZD2, FZD5, FZD7, FZD8	Inhibition of canonical and non-canonical Wnt signaling. Reduces tumor growth and cancer stem cell populations in colorectal, pancreatic, and breast cancers	Gurney et al. (2012), Davis et al. (2020), Diamond et al. (2020)
Vaccine	FZD7 - T7	FZD7	Inhibits tumor development and induces pro-inflammatory cytokine response in triple negative breast cancer mouse model	Hua et al. (2022), Larasati et al. (2022)
Small molecule	LGK974	Wnt	Blocks secretion of Wnt ligands in pancreatic and head and neck cancers	Liu et al. (2013), Rodon et al. (2021)
	SRI3782	FZD7	Targets 7TM domain to inhibit canonical Wnt signaling and cell viability	Zhang et al. (2017)
Decoy receptors	FZD-Fc fusions	Wnt	Sequesters Wnt ligands to reduce Wnt/β-catenin signaling in preclinical cancer studies	Tao et al. (2019), Gurney et al. (2012)
RNA	Small interfering RNAs	FZD7, FZD10	Downregulate overexpressed receptors in colorectal and liver carcinomas	Nagayama et al. (2005), Dang et al. (2024)
	microRNAs (miR-126 and miR-200)	FZD	Suppress expression of FZD interacting partners to attenuate Wnt signaling	Tang et al. (2013), Du et al. (2014)
Gene-editing	CRISPR-Cas9	FZD7	Reduced Wnt/β-catenin signaling in colorectal cancer	Hu et al. (2023)

Small-molecule inhibitors provide another avenue for targeting FZD receptors, either by directly disrupting receptor-ligand interactions or inhibiting associated components of the Wnt pathway. For example, porcupine inhibitors like LGK974 block the secretion of Wnt ligands, indirectly reducing FZD signaling (Liu et al., 2013). These inhibitors have shown promise in preclinical models of Wnt-driven cancers, such as pancreatic, head, and neck cancers (Rodon et al., 2021). Decoy receptors, such as FZD-Fc fusion proteins, mimic the extracellular CRD of FZD receptors, sequestering Wnt ligands to prevent receptor activation (Tao et al., 2019). These fusion proteins have demonstrated efficacy in reducing Wnt/β-catenin signaling in preclinical cancer studies, offering a novel strategy to inhibit aberrant signaling (Gurney et al., 2012). Inhibitors such as SRI3782 have also shown promise for targeting the 7TM region of FZDs, a region typically targeted in other receptors such as class A GPCRs. This molecule, which targets FZD7, introduces a novel approach for inhibition of FZDs by bypassing the highly conserved CRD shared by all class F GPCRs (Zhang et al., 2017).

RNA-based therapies targeting FZD receptors are also under investigation. Small interfering RNAs (siRNAs) have downregulated overexpressed FZD receptors, such as FZD7 and FZD10, in cancers like colorectal and liver carcinomas (Nagayama et al., 2005; Dang et al., 2024). Similarly, microRNA (miRNA) mimics, such as those for miR-126 and miR-200, have shown potential in suppressing FZD interacting partners, such a Wnt and LRP expression, thereby attenuating Wnt signaling and tumor growth (Tang et al., 2013; Du et al., 2014). Emerging technologies like CRISPR-Cas9 offer opportunities to correct mutations in FZD genes or disrupt their overexpression in cancer cells (Steinhart et al., 2017; Han et al., 2024). For example, CRISPR-mediated knockdown of FZD7 has reduced Wnt/β-catenin signaling in preclinical colorectal cancer models, highlighting the potential for gene-editing approaches in targeting FZD-driven oncogenesis (Hu et al., 2023).

Despite these advancements, targeting FZD receptors presents challenges. Wnt signaling plays essential roles in normal tissue homeostasis, including stem cell maintenance, bone formation, and wound healing, making broad inhibition of FZD receptors risky. Tumor heterogeneity and pathway redundancy further complicate therapeutic efforts, as different FZD receptors are overexpressed or functionally dominant across tumor types and stages. Advances in structural biology, such as cryo-electron microscopy, are helping to elucidate FZD receptor-ligand interactions, enabling the development of more selective and context-specific inhibitors. Combination therapies targeting FZD receptors alongside other pathways, such as Notch or Hedgehog, or addressing components of the tumor microenvironment, such as extracellular matrix remodeling, offer potential to enhance therapeutic efficacy while minimizing side effects. Continued research into FZD-targeted therapies is crucial for translating these approaches into clinical success, particularly in cancers driven by dysregulated Wnt signaling.

## Concluding remarks

Frizzled receptors play critical roles in regulating Wnt signaling, a pathway central to cellular processes such as proliferation, migration, and differentiation. In cancer, aberrant FZD signaling drives tumor growth, invasion, metastasis, and therapy resistance, underscoring their importance as therapeutic targets. Advances in structural biology, imaging, and transcriptomics have significantly deepened our understanding of FZD receptor mechanisms, providing a foundation for innovative therapies. Despite these advancements, challenges such as pathway redundancy and tumor heterogeneity must be addressed to optimize therapeutic strategies. Future efforts should focus on developing receptor-specific modulators, refining delivery technologies, and integrating precision medicine to tailor treatments to specific tumor contexts. Continued research into FZD receptor biology holds great promise for advancing cancer therapeutics and improving patient outcomes.
